# Treatment of apathy in stroke patients: a systematic review

**DOI:** 10.3389/fneur.2025.1702325

**Published:** 2025-11-26

**Authors:** Maria Luisa Ruiz-Franco, Laura Amaya-Pascasio, Mercedes Gil-Rodríguez, Antonio Arjona-Padillo, José García-Pinteño, Antonio Jose Rodriguez-Sanchez, Ana Sánchez-Kuhn, Pilar Flores, Patricia Martinez-Sanchez

**Affiliations:** 1 Stroke Unit, Department of Neurology, Torrecárdenas University Hospital, Almería, Spain; 2 Fundation for Biosanitary Research of Eastern Adnalusia (FIBAO), Torrecárdenas University Hospital, Almería, Spain; 3 Department of Psychology, Faculty of Psychology, CTS-280 Clinical and Experimental Neuroscience Research Group and Research Center CiBiS, University of Almeria, Almeria, Spain; 4 Department of Nursing, Physiotherapy and Medicine, Faculty of Health Science, Health Research Center (CEINSA), University of Almería, Almeria, Spain

**Keywords:** apathy, depression, stroke, treatment, post-stroke apathy

## Abstract

**Introduction:**

Post-stroke apathy is a prevalent yet frequently underdiagnosed neuropsychiatric syndrome, reported in up to one-third of stroke survivors, and is consistently associated with poorer functional recovery and cognitive decline. We aimed to review the current evidence on available pharmacological and non-pharmacological treatments for post-stroke apathy, and to evaluate their efficacy and safety.

**Methods:**

A systematic review was conducted following PRISMA guidelines and registered in the PROSPERO database (CRD42022332559). We searched PubMed, Web of Science, and Scopus for randomized and non-randomized clinical trials published until November 2024. Eligible studies included adults with ischemic or hemorrhagic stroke and a defined diagnosis of apathy. Interventions included pharmacological treatments and non-pharmacological strategies, such as neuromodulation techniques. Data extraction and risk of bias assessment were independently performed by two reviewers using the RoB-2 tool.

**Results:**

Ten clinical trials involving 2,359 patients were included. Pharmacological interventions with escitalopram and donepezil (alone or combined with intensive language action therapy) showed potential benefits. Nefiracetam yielded mixed results depending on dose and coexisting depression. Non-pharmacological approaches such as problem-solving therapy, motor relearning programs, strategy training, and complex rehabilitation programs demonstrated significant improvement in apathy scores. High-frequency repetitive transcranial magnetic stimulation also showed efficacy. However, heterogeneity in study design and apathy assessment scales limited direct comparisons.

**Conclusion:**

Several interventions, including escitalopram, donepezil, motor relearning programs, strategy training, and rTMS, have demonstrated potential effectiveness in treating post-stroke apathy. Nevertheless, evidence remains scarce and heterogeneous, underscoring the need for larger, high-quality randomized controlled trials to establish definitive treatment guidelines.

**Systematic review registration:**

https://www.crd.york.ac.uk/PROSPERO/view/CRD42022332559.

## 1 Introduction

Neuropsychiatric disturbances are prevalent sequelae of stroke and significantly compromise functional independence and quality of life. While cognitive impairment and depression have been extensively investigated, apathy remains an under-recognized yet highly prevalent behavioral syndrome in stroke survivors, associated with adverse functional and cognitive outcomes (1).

Post-stroke apathy is particularly common among older individuals and those with prior cerebrovascular pathology (2, 3). Its occurrence appears independent of lesion laterality or stroke subtype, with similar prevalence reported across ischemic and hemorrhagic events (4). Co-occurrence with depression and cognitive deficits is frequent, and both conditions are recognized as significant clinical predictors of apathy (5–7). Although its global impact on clinical outcomes is variable, apathy has been linked to poorer prognosis in younger patients and those with a first-ever stroke (3).

Differentiating apathy from depression remains diagnostically challenging due to overlapping symptomatology, including diminished initiative and reduced goal-directed behavior. However, apathy is characterized by emotional indifference and attenuated affective response, in contrast to the pervasive negative affect that typifies depression (1). Importantly, apathy may also represent an early clinical marker of incipient dementia, including vascular dementia (8). Although its neurobiological substrates are not fully elucidated, converging evidence implicates dysfunction in fronto-subcortical circuits, particularly involving the thalamus, basal ganglia, and prefrontal cortex (1).

The estimated prevalence of post-stroke apathy is approximately 34.6%, rivaling or exceeding that of post-stroke depression (2). However, prevalence estimates vary widely depending on the assessment modality, with higher rates reported in clinician-based evaluations compared to informant- or patient-rated scales (9).

In contrast to other neurodegenerative conditions such as Parkinson’s disease or Alzheimer’s disease, for which apathy management strategies are better established, therapeutic approaches for post-stroke apathy remain poorly defined. Available interventions include selective serotonin reuptake inhibitors, cholinesterase inhibitors, dopaminergic agents, neuropsychological rehabilitation, and non-invasive neuromodulation techniques (1).

Currently, no evidence-based guidelines exist for the clinical management of post-stroke apathy. The present systematic review aims to comprehensively examine the available pharmacological and non-pharmacological treatments for post-stroke apathy and to evaluate their efficacy and safety in this population.

## 2 Methods

### 2.1 Search strategy

This systematic review was conducted in accordance with the Preferred Reporting Items for Systematic Reviews and Meta-Analyses Protocols (PRISMA) guidelines (10) and the protocol was prospectively registered in the PROSPERO database (CRD42022332559). A comprehensive search was performed across PubMed, Web of Science, and Scopus databases for studies published in English or Spanish and indexed in Journal Citation Reports (JCR). The search included literature published up to November 2024.

Eligible studies met the following inclusion criteria: (A) randomized controlled trials (RCTs), quasi-randomized, or non-randomized clinical trials; (B) adult participants (≥18 years) with a diagnosis of ischemic or hemorrhagic stroke; (C) explicit assessment of apathy, with differentiation from depression; and (D) evaluation of a therapeutic intervention targeting apathy, including pharmacological treatments and non-pharmacological strategies such as neuromodulation techniques. Case reports and case series were excluded.

The search strategy combined the following terms:

(“stroke” OR “brain hemorrhage” OR “brain infarction” OR “cerebrovascular disease” OR “cerebral infarction”) AND (“apathy” OR “passivity” OR “indifference” OR “depression” OR “akinetic mutism” OR “anhedonia”) AND (“treatment” OR “antidepressants” OR “non-pharmacological treatment” OR “psychotherapy” OR “neuropsychological advice” OR “neurostimulation” OR “tDCS” OR “TMS” OR “mindfulness”).

Additionally, reference lists of all relevant articles were manually screened to identify further eligible studies.

### 2.2 Study selection

Two independent reviewers (ML R-F and L A-P) screened titles and abstracts for relevance based on predefined eligibility criteria. Duplicate entries were removed using Mendeley and the Rayyan web application (11). Full texts of potentially eligible studies were then reviewed independently to confirm inclusion. Any disagreements were resolved through discussion with a third senior reviewer (P M-S). The study selection process was documented and presented in a PRISMA-compliant flow diagram.

### 2.3 Data extraction

Data extraction was independently performed by two reviewers. Extracted information included study design, population characteristics, intervention details, duration of illness, outcome measures (primary and secondary), and length of follow-up. Discrepancies were resolved through consensus with a third reviewer.

Due to the expected heterogeneity in study design, populations, and outcome measures, a quantitative meta-analysis was not performed.

### 2.4 Risk of bias assessment

The risk of bias of included studies was evaluated using the Cochrane Risk of Bias tool version 2.0 (RoB-2) (12), which assesses five domains: (A) bias arising from the randomization process, (B) deviations from intended interventions, (C) missing outcome data, (D) outcome measurement, and (E) selection of the reported result. Risk levels were categorized as follows: “high risk” if at least one domain was rated high; “some concerns” if one or more domains raised concerns but none were rated high; and “low risk” if all domains were rated as low risk.

## 3 Results

**Figure 1:**
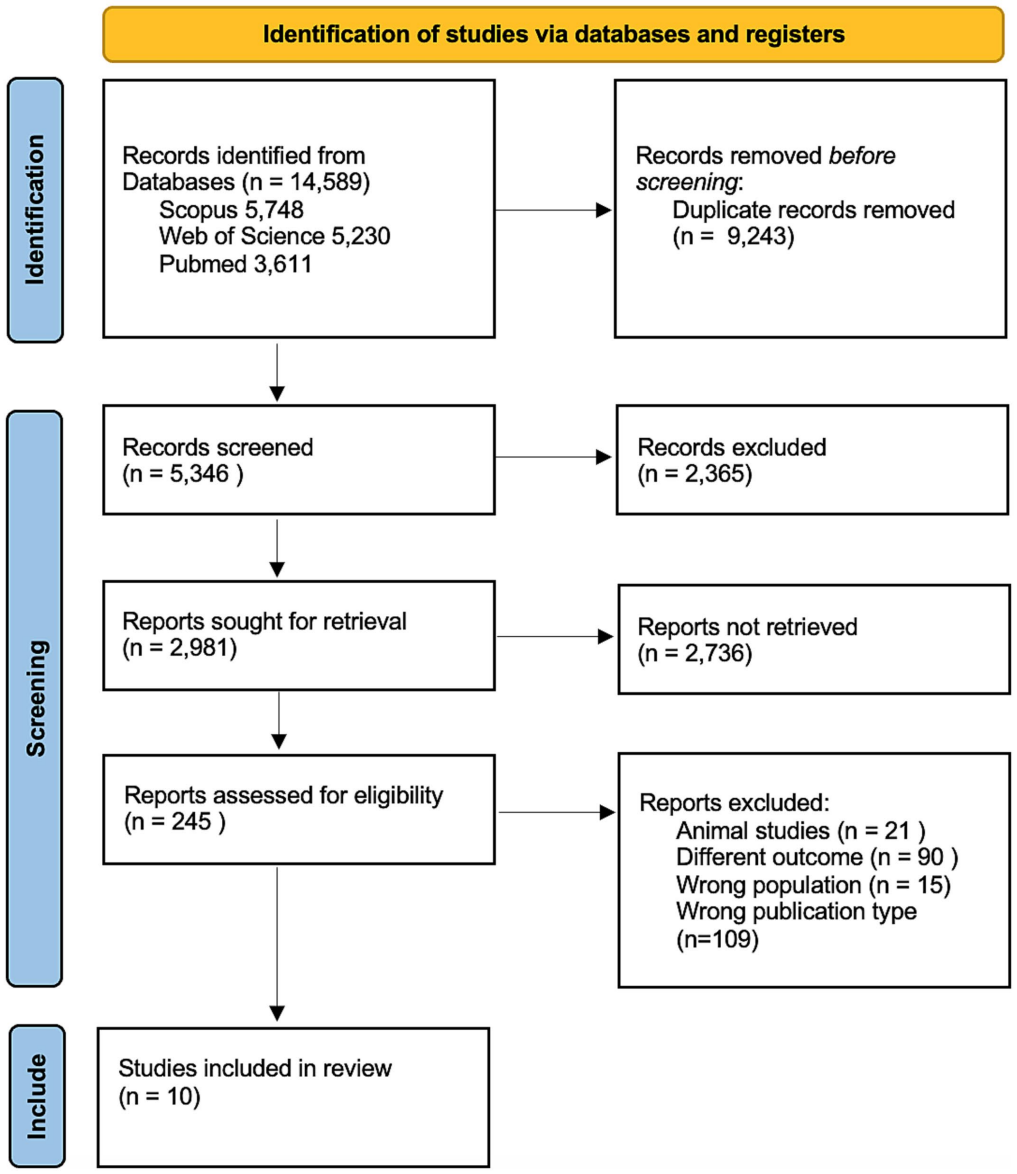
PRISMA flow diagram.

Following removal of duplicates, a total of 5,346 records were identified through database searches and screened for eligibility. Of these, 245 full-text articles were assessed, and 10 studies met the inclusion criteria for this systematic review. The study selection process is detailed in the PRISMA flow diagram (Figure 1).

### 3.1 Study characteristics

A total of 10 studies encompassing 2,359 participants were included in the final analysis. Among these, 8 were randomized controlled trials (RCTs) and 2 were open-label trials. Key study characteristics are summarized in Table 1. The mean age of participants ranged from 38 to 80 years, with a predominance of male subjects. Lesion distribution was heterogeneous, involving multiple cerebrovascular territories.

**Table 1:** Clinical trial characteristics.

Author, study type	Population mean age/male	Stroke type	Apathy diagnosis	Intervention	Comparator	Outcome
Pharmocological interventions						
Whyte et al. (15) Open-label study	13 69.1/8	Ischemic	AES	Donepezil, Galantamine	Historical control group	Improvement of AES punctuation
Robinson et al. (16). RCT	70 66.3/40	Ischemic/hemorrhagic	Apathy Scale	Nefiracetam (600 or 900 mg/day)	Placebo	Nefiracetam significantly improved apathy scores
Mikami et al. (13) RCT	154 63.7/93	Ischemic/hemorrhagic (<3 months)	AES	Escitalopram, PST	Placebo	Effective to prevent apathy onset, lower risk with escitalopram and PST.
Starkstein et al. (17) RCT	13 68,65/10	Ischemic/hemorrhagic	Apathy Scale	Nefiracetam (900 mg/day)	Placebo	No significant improvement in apathy scores
Tay et al. (18). RCT (Post-hoc)	1,369 70.7/846	Ischemic/hemorrhagic	MADRS	Fluoxetine	Placebo	Apathetic scores No reduction in apthy. Fluoxetine reduced depression.
Berthier et al. (14) Open label-study	10 51.6/8	Chronic ischemic/ hemorrhagic	SADQ-21	Donepezil + ILAT	Donepezil alone	Donepezil-ILAT improved apathy and language outcomes
Non-pharmacological interventions						
Skidmore et al. (21). RCT	30 68.33/20	Acute ischemic/hemorrhagic	AES	Strategy training	Reflective listening	Strategy training was associated with significantly lower levels of post-stroke apathy.
Mayo et al. (22). RCT	186 63/113	Ischemic/hemorrhagic	Apathy Scale	Immediate group-based rehabilitation program	Delayed group-based rehabilitation program.	Both inmmediate and delayed interventions significantly increased meaningful activity and reduced apathy
Sasaki et al. (23) RCT	13 64.45/11	Chronic ischemic/hemorrhagic	Apathy Scale	Hugh-frequency rTMS	Sham stimulation	rTMS significantly improved apathy aymptoms.
Chen et al. (19). RCT	488 65.61/258	Ischemic	AES	MRP	Bobath approach	MRP was significantly more effective in preventing of new onset of apathy following stroke

*AES, Apathy evaluation scale; PST, problem-solving therapy; SADQ-21, Apathy and depression subdomain scores of the Stroke Aphasia Depression Questionnaire-21; ILAT, intensive language action therapy; MADRS, Montgomery-Asberg Depression Rating Scale MRP, motor relearning program; RCT, Randomized clinical trial.

### 3.2 Risk of bias assessment

**Figure 2:**
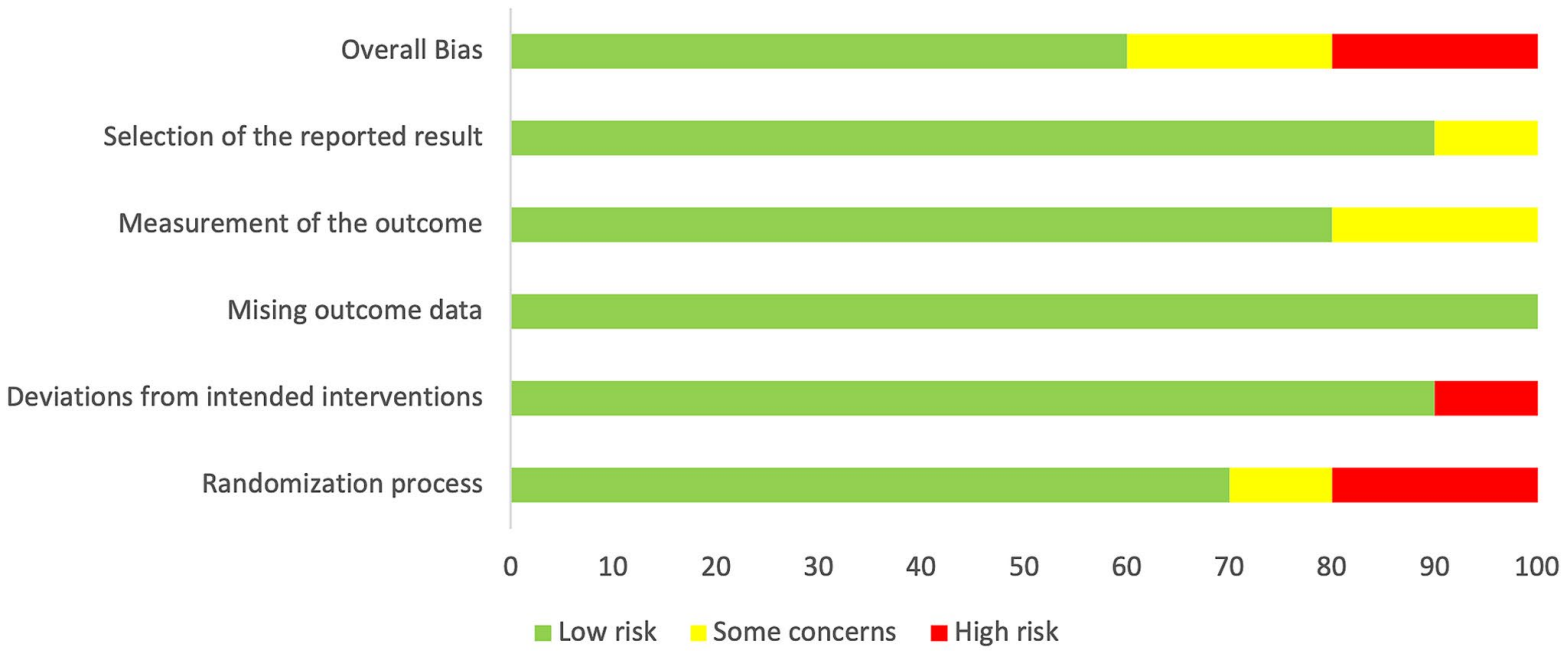
Summary of the quality of the included studies according to the RoB-2 tool.

**Figure 3:**
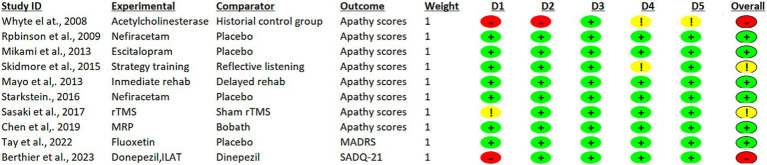
Assessment of risk of bias: RoB-2 tool to evaluate the methodological quality of randomized crossover studies and randomized clinical trials (RCT).

The certainty of the evidence presented in the studies was evaluated in the Rob 2 tool and is summarized in Figure 2. Six RCTs had a low RoB 2, two had high Rob2, and two had some concerns (Figure 3).

### 3.3 Pharmacological treatment

#### 3.3.1 Escitalopram

Mikami et al. (13) demonstrated a preventive effect of escitalopram on the development of post-stroke apathy in patients enrolled within the first 3 months following the cerebrovascular event. In this randomized controlled trial involving 154 participants, subjects were assigned to receive escitalopram, placebo, or problem-solving therapy (PST). Kaplan–Meier analysis revealed that individuals in the placebo group were 3.47 times more likely to develop apathy compared to those treated with escitalopram (*p* < 0.0001). No significant differences in the incidence of adverse events were observed between groups.

#### 3.3.2 Acetylcholinesterase inhibitors (donepezil and galantamine)

Berthier et al. (14) conducted a 10-week open-label feasibility study in 10 patients with chronic post-stroke aphasia following left perisylvian stroke. Participants received donepezil 5 mg/day for 4 weeks, followed by 10 mg/day for another 4 weeks, and finally donepezil 10 mg/day combined with 3 h per day of intensive language action therapy (ILAT) for 2 weeks. Apathy and depression were assessed using the relevant subscales of the Stroke Aphasia Depression Questionnaire–21 (SADQ-21), along with the Western Aphasia Battery–Aphasia Quotient (WAB-AQ). Significant improvements in apathy were observed only following the combined donepezil + ILAT intervention (*p* = 0.007), but not with donepezil alone (*p* = 0.125), nor when comparing donepezil monotherapy to the combined approach (*p* = 0.203). Depression scores also improved significantly with the combined therapy versus donepezil alone (*p* = 0.023).

Whyte et al. (15) evaluated the impact of acetylcholinesterase inhibitors on cognitive and functional recovery in an open-label pilot study of stroke patients over 60 years old with post-stroke cognitive decline (excluding individuals with prior Alzheimer’s disease). Patients received either donepezil or galantamine for 12 weeks. AES scores improved progressively throughout the intervention. Notably, in the donepezil group, changes in AES scores were positively associated with gains in Functional Independence Measure–motor subscale scores. However, given that a historical control group was used and apathy was not systematically assessed in controls, direct conclusions regarding treatment efficacy for apathy could not be drawn.

#### 3.3.3 Nefiracetam

The effectiveness of nefiracetam in post-stroke apathy has been assessed in two randomized trials with conflicting findings. Robinson et al. (16) included 70 patients with major depressive disorder and coexisting apathy 3 months post-stroke. Participants were randomized to receive 900 mg/day or 600 mg/day of nefiracetam, or placebo. The 900 mg/day group exhibited significantly greater reductions in Apathy Scale (AS) scores compared to both the lower-dose and placebo groups (*p* = 0.05), without differences in depressive symptoms. In contrast, Starkstein et al. (17) studied 13 non-depressed patients with apathy within 2 months post-stroke, randomized to 450 mg/day of nefiracetam or placebo. While AS scores declined by an average of 7 points in the nefiracetam group, this difference was not statistically significant. Variability in sample size, dose, and patient characteristics likely contributed to the inconsistent results, precluding firm conclusions.

#### 3.3.4 Fluoxetine

Tay et al. (18) assessed the efficacy of fluoxetine for post-stroke apathy in a randomized trial involving 1,369 patients assigned to fluoxetine (*n* = 681) or placebo (*n* = 688) for 6 months. The proportion of patients exhibiting apathy remained unchanged in the fluoxetine group, while it increased in the placebo group. However, both groups demonstrated a significant increase in apathy symptom scores over time, suggesting limited clinical benefit.

### 3.4 Non-pharmacological treatment

#### 3.4.1 Problem-solving therapy

In the same trial by Mikami et al. (13), PST—a structured intervention designed to enhance adaptive coping and goal-directed behavior—was associated with a significantly reduced risk of post-stroke apathy compared to placebo. After adjusting for confounding variables (age, sex, cognitive status, diabetes), patients in the placebo group were 1.84 times more likely to develop apathy. The protective effect of PST reached statistical significance.

#### 3.4.2 Motor relearning program

MRP is a rehabilitation strategy that emphasizes active patient engagement in functional task practice. Chen et al. (19) compared MRP to the Bobath approach in a randomized controlled trial including 488 patients with first-ever ischemic stroke within 7 days of onset, none of whom met apathy criteria at baseline. The main difference between the Motor Relearning Program (MRP) and the Bobath approach lies in their underlying principles: while Bobath emphasizes therapist-guided facilitation of normal movement patterns, MRP is based on motor learning theory and promotes active problem solving and patient participation in functional tasks. Given that apathy is closely related to reduced initiative and motivation, MRP may be more effective in preventing poststroke apathy by actively engaging patients in goal-directed activities and enhancing motivational drive. Patients were randomized to receive either MRP (*n* = 245) or Bobath-based therapy (*n* = 243), with a 12-month follow-up. Although AES scores declined in both groups, apathy severity was significantly lower in the MRP group. Moreover, individuals in the Bobath group were 1.63 times more likely to develop apathy compared to those in the MRP group.

#### 3.4.3 Strategy training

Strategy training incorporates techniques such as goal setting, self-monitoring, and problem-solving to foster autonomy and engagement in rehabilitation. While previous studies have shown benefits for cognitive and functional recovery, its effect on apathy had not been specifically explored (20). Skidmore et al. (21) randomized stroke patients with cognitive impairment to receive either strategy training or reflective listening, in addition to standard inpatient rehabilitation. Over a six-month follow-up, the strategy training group exhibited significantly lower apathy scores at 3 months, with a moderate-to-large, statistically significant effect. At 6 months, the reduction in apathy persisted, though without statistical significance. In contrast, apathy symptoms increased over time in the control group.

#### 3.4.4 Complex intervention

Mayo et al. (22) evaluated a structured, community-based rehabilitation program designed with direct input from stroke survivors. Patients within 5 years post-stroke were randomized to immediate or delayed program initiation (4 months). Participants attended group sessions held in a community-based setting twice per week, each lasting approximately 3 h, organized into three consecutive 3-month blocks, for a total program duration of 12 months. Although the intervention significantly reduced apathy levels, the between-group difference did not exceed the threshold for clinical significance.

#### 3.4.5 Repetitive transcranial magnetic stimulation

Sasaki et al. (23) examined the therapeutic potential of high-frequency rTMS in 13 patients with chronic post-stroke apathy. Participants were randomized to receive active or sham stimulation. They received 5 sessions of either rTMS or sham stimulation over 5 consecutive days. The rTMS protocol targeted the dorsal anterior cingulate and medial prefrontal cortex using 10 Hz trains (10 s on, 50 s off; 2,000 pulses per session, 20 min total). The active rTMS group showed a significantly greater reduction in AES scores compared to the sham group, suggesting that neuromodulation may improve motivational deficits in this population.

## 4 Discussion

To our knowledge, this is the first systematic review specifically focused on the treatment of post-stroke apathy that comprehensively evaluates both pharmacological and non-pharmacological interventions in adult stroke survivors. Although apathy is one of the most prevalent neuropsychiatric sequelae of stroke, it remains underdiagnosed and poorly managed in clinical practice. Our findings integrate pharmacological and non-pharmacological interventions and highlight promising candidates such as escitalopram, galantamine, donepezil (alone or in combination with ILAT), motor relearning programs (MRP), strategy training, community-based interventions (EBP), and rTMS. Nevertheless, the substantial methodological heterogeneity across studies and the overall scarcity of robust data underscores the need for high-quality randomized trials.

Apathy, characterized by a marked reduction in goal-directed behavior, affects approximately one-third of stroke survivors and is consistently associated with worse functional and cognitive outcomes (1). Despite its clinical significance, apathy is often overshadowed by post-stroke depression and cognitive impairment. Importantly, it is not yet formally recognized as a clinical syndrome in major diagnostic systems. While the ICD-11 classifies apathy as a symptom under code MB24.4, and the DSM-5 includes it as a feature of other disorders (e.g., mood or neurocognitive syndromes), neither system defines it as a standalone diagnosis (24).

This lack of formal nosological status limits clinical recognition and research. Diagnosis relies instead on various operationalized criteria and rating scales. While tools such as the Neuropsychiatric Inventory (NPI) include apathy as part of a broader syndrome (25), several instruments have been specifically developed to measure apathy: the Structured Clinical Interview for Apathy (SCIA) (26), Apathy Evaluation Scale (AES) (27), Apathy Scale (AS) (28), Apathy Inventory (AI) (29), Dementia Apathy Interview and Rating (DAIR) (30), and Lille Apathy Rating Scale (LARS) (31), many of which are available in validated Spanish versions.

Neurobiologically, apathy is thought to result from dysfunction in brain networks involving the prefrontal cortex, basal ganglia, and their interconnecting circuits, modulated by neurotransmitters such as dopamine, serotonin, acetylcholine, and norepinephrine (32). Based on lesion studies, Levy and Dubois have proposed three apathy subtypes—emotional-affective, cognitive, and auto-activation deficit—each linked to distinct disruptions in fronto-subcortical loops (3, 33).

Despite the high prevalence and clinical impact of post-stroke apathy, interventional research remains limited. Most studies rely on the AES for diagnosis and follow-up. Escitalopram (13), galantamine (15), donepezil monotherapy (15), and donepezil combined with ILAT (14) were associated with improvements in apathy measures. Nefiracetam also showed beneficial effects in one trial involving patients with comorbid depression (16) but failed to demonstrate efficacy in non-depressed individuals (17).

Among non-pharmacological strategies, MRP (19), strategy training (21), EBP (22), and rTMS (23) demonstrated improvements in apathy outcomes. However, small sample sizes and varied methodologies limit generalizability.

Management of apathy is better characterized in other neurological disorders. Apathy is highly prevalent in dementia, particularly Alzheimer’s disease, where it is linked to accelerated functional and cognitive decline (34). Despite this, no pharmacological treatment is currently approved for apathy in any condition. Acetylcholinesterase inhibitors have demonstrated some efficacy in Alzheimer’s (34) and Parkinson’s disease (35). Rivastigmine improved apathy in dementia with Lewy bodies (36), while memantine evidence is less consistent (37).

Psychostimulants, particularly methylphenidate, have shown efficacy in Alzheimer’s (38, 39) and Parkinson’s disease (40), either as monotherapy or combined with cholinesterase inhibitors. Several case reports suggest potential benefit in post-stroke apathy as well (41, 42). On the other hand, serotoninergic antidepressants have not proven effective for apathy in the absence of major depression, particularly in Alzheimer’s disease (43–46). In frontotemporal dementia, agomelatine has shown promise in a single trial (47).

Non-pharmacological interventions such as music therapy, individualized engagement, cognitive stimulation, multisensory behavioral therapy, art therapy, and therapeutic conversation are recommended in international guidelines (48), although their evidence base in stroke populations is limited.

There are also case reports describing improvement of post-stroke apathy with other agents: bromocriptine in two patients with lacunar infarcts (49, 50), zolpidem in a patient with a right hemispheric hemorrhagic stroke (51), and olanzapine in a patient with a left MCA infarction (52). While intriguing, these observations remain anecdotal and warrant further controlled investigation.

This review has some limitations. The number of eligible trials was small, with many studies being underpowered, lacking blinding, small sample or using heterogeneous designs in terms of apathy definitions, outcome measures (apathy scores), and timing of intervention. The use of apathy subscales within broader depression tools may have limited diagnostic specificity, and the absence of meta-analysis prevents quantitative synthesis. Moreover, comparability across trials is limited by the variability in apathy assessment tools. Recent studies (53, 54) have developed multidimensional instruments for evaluating this condition, which may help in the future to address differences arising from the diverse cultural and clinical contexts in which the various trials are conducted. Nonetheless, this is the first systematic review to examine both pharmacological and nonpharmacological interventions for post-stroke apathy, applying rigorous methodology, predefined inclusion criteria, a comprehensive search strategy, and independent bias assessment, enhancing its reliability and clinical relevance.

## 5 Conclusion

Several therapeutic strategies—including escitalopram, galantamine, donepezil (alone or with ILAT), MRP, strategy training, EBP, and rTMS—have shown potential efficacy in clinical trials for post-stroke apathy. However, the evidence base remains limited, heterogeneous, and based largely on small samples. These findings highlight the urgent need for large-scale, multicenter, randomized controlled trials with standardized diagnostic criteria and outcome measures to establish evidence-based recommendations for the management of post-stroke apathy.

## Data Availability

The original contributions presented in the study are included in the article/supplementary material, further inquiries can be directed to the corresponding authors.

## References

[1] Tay J Morris RG Markus HS. Apathy after stroke: diagnosis, mechanisms, consequences, and treatment. Int J Stroke. (2021) 16:510–8. doi: 10.1177/1747493021990906, PMID: 33527880 PMC8267086

[2] Caeiro L Ferro JM Costa J. Apathy secondary to stroke: a systematic review and Meta-analysis. Cerebrovasc Dis. (2013) 35:23–39. doi: 10.1159/000346076, PMID: 23428994

[3] Marin RS. Apathy: a neuropsychiatric syndrome. J Neuropsychiatry Clin Neurosci. (1991) 3:243–54. doi: 10.1176/jnp.3.3.243, PMID: 1821241

[4] Singh M Cameron J. Psychosocial aspects of caregiving to stroke patients. Axone. (2005) 27:18–24. doi: 10.1111/j.1365-2648.2005.03551.x PMID: 16259231

[5] Caeiro L Ferro JM Figueira ML. Apathy in acute stroke patients. Eur J Neurol. (2012) 19:291–7. doi: 10.1111/j.1468-1331.2011.03508.x, PMID: 21895880

[6] Onyike CU Sheppard JM Tschanz JT Norton MC Green RC Steinberg M . Epidemiology of apathy in older adults: the cache county study. Am J Geriatr Psychiatry. (2007) 15:365–75. doi: 10.1097/01.JGP.0000235689.42910.0d 17463187

[7] Brodaty H Altendorf A Withall A Sachdev P. Do people become more apathetic as they grow older? A longitudinal study in healthy individuals. Int Psychogeriatr. (2010) 22:426–36. doi: 10.1017/S1041610209991335, PMID: 20003630

[8] Tay J Morris RG Tuladhar AM Husain M De Leeuw FE Markus HS. Apathy, but not depression, predicts all-cause dementia in cerebral small vessel disease. J Neurol Neurosurg Psychiatry. (2020) 91:953–9. doi: 10.1136/jnnp-2020-323092, PMID: 32651249 PMC7476304

[9] Van Dalen JW Van Charante EPM Nederkoorn PJ Van Gool WA Richard E. Poststroke apathy. Stroke. (2013) 44:851–60. doi: 10.1161/STROKEAHA.112.674614, PMID: 23362076

[10] Page MJ McKenzie JE Bossuyt PM Boutron I Hoffmann TC Mulrow CD . The PRISMA 2020 statement: an updated guideline for reporting systematic reviews. Syst Rev. (2021) 10:89. doi: 10.1186/s13643-021-01626-4, PMID: 33781348 PMC8008539

[11] Ouzzani M Hammady H Fedorowicz Z Elmagarmid A. Rayyan web and mobile app for systematic reviews. Syst Rev. (2016) 5:210. doi: 10.1186/s13643-016-0384-4 27919275 PMC5139140

[12] Sterne JAC Savović J Page MJ Elbers RG Blencowe NS Boutron I . Rob 2: a revised tool for assessing the risk of bias in randomized trials. BMJ. (2019) 366:1–8. doi: 10.1136/bmj.l4898 31462531

[13] Mikami K Jorge RE Moser DJ Arndt S Jang M Solodkin A . Prevention of poststroke apathy using escitalopram or problem-solving therapy. Am J Geriatr Psychiatry. (2013) 21:855–62. doi: 10.1016/j.jagp.2012.07.003, PMID: 23930743

[14] Berthier ML Edelkraut L López-González FJ López-Barroso D Mohr B Pulvermüller F . Donepezil alone and combined with intensive language-action therapy on depression and apathy in chronic post-stroke aphasia: a feasibility study. Brain Lang. (2023) 236:105205. doi: 10.1016/j.bandl.2022.105205, PMID: 36495749

[15] Whyte EM Lenze EJ Butters M Skidmore E Koenig K Dew MA . An open-label pilot study of acetylcholinesterase inhibitors to promote functional recovery in elderly cognitively impaired stroke patients. Cerebrovasc Dis. (2008) 26:317–21. doi: 10.1159/000149580, PMID: 18667813 PMC2914451

[16] Robinson RG Jorge RE Clarence-Smith K Starkstein S. Double-blind treatment of apathy in patients with poststroke depression using nefiracetam. J Neuropsychiatry Clin Neurosci. (2009) 21:144–51. doi: 10.1176/jnp.2009.21.2.144, PMID: 19622685

[17] Starkstein SE Brockman S Hatch KK Bruce DG Almeida OP Davis WA . A randomized, placebo-controlled, double-blind efficacy study of Nefiracetam to treat Poststroke apathy. J Stroke Cerebrovasc Dis. (2016) 25:1119–27. doi: 10.1016/j.jstrokecerebrovasdis.2016.01.032, PMID: 26915605

[18] Tay J EFFECTS Trial Collaboration Mårtensson B Markus HS Lundström E. Does fluoxetine reduce apathetic and depressive symptoms after stroke? An analysis of the efficacy of fluoxetine randomized controlled trial in stroke trial data set. Int J Stroke. (2023) 18:285–95. doi: 10.1177/17474930221124760, PMID: 36050815 PMC9940155

[19] Chen L Xiong S Liu Y Lin M Zhu L Zhong R . Comparison of motor relearning program versus Bobath approach for prevention of Poststroke apathy: a randomized controlled trial. J Stroke Cerebrovasc Dis. (2019) 28:655–64. doi: 10.1016/j.jstrokecerebrovasdis.2018.11.011, PMID: 30501977

[20] Skidmore ER Whyte EM Butters MA Terhorst L Reynolds CF III. Strategy training during inpatient rehabilitation may prevent apathy symptoms after acute stroke. PMR. (2015) 7:562–70. doi: 10.1016/j.pmrj.2014.12.010, PMID: 25595665 PMC4466065

[21] Skidmore ER Dawson DR Butters MA Dew MA Grattan ES Juengst SB . Strategy training shows promise for addressing disability in the first 6 months after a stroke. Neurorehabil Neural Repair. (2015) 29:668–76. doi: 10.1177/1545968314562113 25505221 PMC4465421

[22] Mayo NE Anderson S Barclay R Cameron JI Desrosiers J Eng JJ . Getting on with the rest of your life following stroke: a randomized trial of a complex intervention aimed at enhancing life participation post-stroke. Clin Rehabil. (2015) 29:1198–211. doi: 10.1177/0269215514565396, PMID: 25627292

[23] Sasaki N Hara T Yamada N Niimi M Kakuda W Abo M. The efficacy of high frequency repetitive transcranial magnetic stimulation for improving apathy in chronic stroke patients. Eur Neurol. (2017) 78:28–32. doi: 10.1159/000477440, PMID: 28578330

[24] American Psychiatric Association. Diagnostic and statistical manual of mental disorders: DSM-5. 5th ed. Washington, DC: American Psychiatric Association (2013).

[25] Cummings JL Mega M Gray K Rosenberg-Thompson S Carusi DA Gornbein J. The neuropsychiatric inventory: comprehensive assessment of psychopathology in dementia. Neurology. (1994) 44:2308–2308. doi: 10.1212/WNL.44.12.2308, PMID: 7991117

[26] Starkstein SE Ingram L Garau ML Mizrahi R. On the overlap between apathy and depression in dementia. J Neurol Neurosurg Psychiatry. (2005) 76:1070–4. doi: 10.1136/jnnp.2004.052795, PMID: 16024880 PMC1739766

[27] Marin RS Biedrzycki RC Firinciogullari S. Reliability and validity of the apathy evaluation scale. Psychiatry Res. (1991) 38:143–62. doi: 10.1016/0165-1781(91)90040-v, PMID: 1754629

[28] Starkstein SE Mayberg HS Preziosi TJ Andrezejewski P Leiguarda R Robinson RG. Reliability, validity and clinical correlates of apathy in Parkinson's disease. J Neuropsychiatry Clin Neurosci. (1992) 4:134–9. doi: 10.1176/jnp.4.2.134, PMID: 1627973

[29] Robert PH Clairet S Benoit M Koutaich J Bertogliati C Tible O . The apathy inventory: assessment of apathy and awareness in Alzheimer's disease, Parkinson's disease and mild cognitive impairment. Int J Geriatr Psychiatry. (2002) 17:1099–105. doi: 10.1002/gps.755 12461757

[30] Strauss ME Sperry SD. An informant-based assessment of apathy in Alzheimer disease. Neuropsychiatry Neuropsychol Behav Neurol. (2002) 15:176–83. doi: 10.1097/01.WNN.0000020010.79870.C3 12218710

[31] Sockeel P Dujardin K Devos D Denève C Destée A Defebvre L. The Lille apathy rating scale (LARS), a new instrument for detecting and quantifying apathy: validation in Parkinson's disease. J Neurol Neurosurg Psychiatry. (2006) 77:579–84. doi: 10.1136/jnnp.2005.075929, PMID: 16614016 PMC2117430

[32] López-Dóriga Bonnardeaux P Andrino Díaz N. Apatía postictus [post-stroke apathy]. Rev Esp Geriatr Gerontol. (2016) 51:164–9. doi: 10.1016/j.regg.2015.09.002 26522489

[33] Levy R Czernecki V. Apathy and the basal ganglia. J Neurol. (2006) 253:VII54–61. doi: 10.1007/s00415-006-7012-5, PMID: 17131230

[34] Azhar L Kusumo RW Marotta G Lanctôt KL Herrmann N. Pharmacological management of apathy in dementia. CNS Drugs. (2022) 36:143–65. doi: 10.1007/s40263-021-00883-0, PMID: 35006557

[35] Devos D Moreau C Maltête D Lefaucheur R Kreisler A Eusebio A . Rivastigmine in apathetic but dementia and depression-free patients with Parkinson’s disease: a double-blind, placebo-controlled, randomised clinical trial. J Neurol Neurosurg Psychiatry. (2014) 85:668–74. doi: 10.1136/jnnp-2013-306439, PMID: 24218528

[36] McKeith I Del Ser T Spano P Emre M Wesnes K Anand R . Efficacy of rivastigmine in dementia with Lewy bodies: a randomised, double-blind, placebo-controlled international study. Lancet. (2000) 356:2031–6. doi: 10.1016/S0140-6736(00)03399-7 11145488

[37] Gauthier S Loft H Cummings J. Improvement in behavioural symptoms in patients with moderate to severe Alzheimer’s disease by memantine: a pooled data analysis. Int J Geriatr Psychiatry. (2008) 23:537–45. doi: 10.1002/gps.1949, PMID: 18058838

[38] Mintzer J Lanctôt KL Scherer RW Rosenberg PB Herrmann N van Dyck CH . Effect of methylphenidate on apathy in patients with Alzheimer disease: the ADMET 2 randomized clinical trial. JAMA Neurol. (2021) 78:1324–32. doi: 10.1001/jamaneurol.2021.3356, PMID: 34570180 PMC8477302

[39] Ruthirakuhan MT Herrmann N Abraham EH Chan S Lanctôt KL. Pharmacological interventions for apathy in Alzheimer's disease. Cochrane Database Syst Rev. (2018) 5:CD012197. doi: 10.1002/14651858.CD012197 29727467 PMC6494556

[40] Moreau C Delval A Defebvre L Dujardin K Duhamel A Petyt G . Methylphenidate for gait hypokinesia and freezing in patients with Parkinson’s disease undergoing subthalamic stimulation: a multicentre, parallel, randomised, placebo-controlled trial. Lancet Neurol. (2012) 11:589–96. doi: 10.1016/S1474-4422(12)70106-0, PMID: 22658702

[41] Watanabe MD Martin EM DeLeon OA Gaviria M Pavel DG Trepashko DW. Successful methylphenidate treatment of apathy after subcortical infarcts. J Neuropsychiatry Clin Neurosci. (1995) 7:502–4. doi: 10.1176/jnp.7.4.502, PMID: 8555754

[42] Spiegel DR Kim J Greene K Conner C Zamfir D. Apathy due to cerebrovascular accidents successfully treated with methylphenidate: a case series. J Neuropsychiatry Clin Neurosci. (2009) 21:216–9. doi: 10.1176/jnp.2009.21.2.216, PMID: 19622693

[43] Lawlor BA Sunderland T Mellow AM Hill JL Molchan SE Murphy DL. Hyperresponsivity to the serotonin agonist m-chlorophenylpiperazine in Alzheimer’s disease. A controlled study. Arch Gen Psychiatry. (1989) 46:542–9. doi: 10.1001/archpsyc.1989.01810060064010 2730278

[44] Lanctôt KL Herrmann N van Reekum R Eryavec G Naranjo CA. Gender, aggression and serotonergic function are associated with response to sertraline for behavioral disturbances in Alzheimer’s disease. Int J Geriatr Psychiatry. (2002) 17:531–41. doi: 10.1002/gps.636, PMID: 12112177

[45] Zhou T Wang J Xin C Kong L Wang C. Effect of memantine combined with citalopram on cognition of BPSD and moderate Alzheimer’s disease: a clinical trial. Exp Ther Med. (2019) 17:1625–30. doi: 10.3892/etm.2018.7124, PMID: 30783429 PMC6364245

[46] Leonpacher AK Peters ME Drye LT Makino KM Newell JA Devanand DP . Effects of citalopram on neuropsychiatric symptoms in Alzheimer’s dementia: evidence from the CitAD study. Am J Psychiatry. (2016) 173:473–80. doi: 10.1176/appi.ajp.2016.15020248, PMID: 27032628

[47] Callegari I Mattei C Benassi F Krueger F Grafman J Yaldizli Ö . Agomelatine improves apathy in frontotemporal dementia. Neurodegener Dis. (2016) 16:352–6. doi: 10.1159/000445873, PMID: 27229348

[48] Cai Y Li L Xu C Wang Z. The effectiveness of non-pharmacological interventions on apathy in patients with dementia: a systematic review of systematic reviews. Worldviews Evid-Based Nurs. (2020) 17:311–8. doi: 10.1111/wvn.12459, PMID: 32767834

[49] Parks RW Crockett DJ Manji HK Ammann W. Assessment of bromocriptine intervention for the treatment of frontal lobe syndrome: a case study. J Neuropsychiatry Clin Neurosci. (1992) 4:109–11. doi: 10.1176/jnp.4.1.109. PMID: 1627954, PMID: 1627954

[50] Barrett K. Treating organic abulia with bromocriptine and lisuride: four case studies. J Neurol Neurosurg Psychiatry. (1991) 54:718–21. doi: 10.1136/jnnp.54.8.718. PMID: 1940945, PMID: 1940945 PMC1014478

[51] Mathieu S Autret K Arnould A Travers C Charveriat S Vandenhelsken C . Treatment of apathy with zolpidem (Stilnox): two double- blind, placebo-controlled single case studies. Ann Phys Rehabil Med. (2011) 54:e214. doi: 10.1016/j.rehab.2011.07.389

[52] Connor AT Crawford A Levy RJ Schneider LM Hollander SA Shaw RJ. A case of abulia from left middle cerebral artery stroke in an adolescent treated successfully with short duration olanzapine. Clin Neuropharmacol. (2020) 43:86–9. doi: 10.1097/WNF.0000000000000389, PMID: 32384311

[53] Lee B Gleason C Umucu E. Clinical utility and psychometric properties of the apathy evaluation scale. Rehabil Psychol. (2020) 65:311–2. doi: 10.1037/rep0000356, PMID: 32804534 PMC8127218

[54] Yi HJ Tan CH Hong WP Yu RL. Development and validation of the geriatric apathy scale: examining multi-dimensional apathy profiles in a neurodegenerative population with cultural considerations. Asian J Psychiatr. (2024) 93:103924. doi: 10.1016/j.ajp.2024.103924, PMID: 38232445

